# Estimating PM_2.5_ Concentrations Based on MODIS AOD and NAQPMS Data over Beijing–Tianjin–Hebei

**DOI:** 10.3390/s19051207

**Published:** 2019-03-09

**Authors:** Qingxin Wang, Qiaolin Zeng, Jinhua Tao, Lin Sun, Liang Zhang, Tianyu Gu, Zifeng Wang, Liangfu Chen

**Affiliations:** 1 College of Geomatics, Shandong University of Science and Technology, Qingdao 266590, China; wangqingxin_rs@163.com (Q.W.); Sunlin6@126.com (L.S.); 2 State Key Laboratory of Remote Sensing Science, Institute of Remote Sensing and Digital Earth of Chinese Academy of Sciences, Beijing 100101, China; wangzf@radi.ac.cn (Z.W.); Chenlf@radi.ac.cn (L.C.); 3 University of Chinese Academy of Sciences, Beijing 100049, China; 4 Chongqing Engineering Research Center of Spatial Big Data Intelligent Technology, Chongqing University of Posts and Telecommunications, Chongqing 400065, China; 5 Environmental Emergency and Heavy Pollution Weather Warning Center, Shijiazhuang 050000, China; 8807262008@163.com (L.Z.); gty0707@163.com (T.G.)

**Keywords:** PM_2.5_, MODIS AOD, NAQPMS, LME, BTH

## Abstract

Accurately estimating fine ambient particulate matter (PM_2.5_) is important to assess air quality and to support epidemiological studies. To analyze the spatiotemporal variation of PM_2.5_ concentrations, previous studies used different methodologies, such as statistical models or neural networks, to estimate PM_2.5_. However, there is little research on full-coverage PM_2.5_ estimation using a combination of ground-measured, satellite-estimated, and atmospheric chemical model data. In this study, the linear mixed effect (LME) model, which used the aerosol optical depth (AOD) from the Moderate Resolution Imaging Spectroradiometer (MODIS), meteorological data, normalized difference vegetation index (NDVI), and elevation data as predictors, was fitted for 2017 over Beijing–Tianjin–Hebei (BTH). The LME model was used to calibrate the PM_2.5_ concentration using the nested air-quality prediction modeling system (NAQPMS) simulated with ground measurements. The inverse variance weighting (IVW) method was used to fuse satellite-estimated and model-calibrated PM_2.5_. The results showed a strong agreement with ground measurements, with an overall coefficient (*R*^2^) of 0.78 and a root-mean-square error (RMSE) of 26.44 μg/m^3^ in cross-validation (CV). The seasonal *R*^2^ values were 0.75, 0.62, 0.80, and 0.78 in the spring, summer, autumn, and winter, respectively. The fusion results supplement the lack of satellite estimates and can capture more detailed information than the NAQPMS model. Therefore, the results will be helpful for pollution process analyses and health-related studies.

## 1. Introduction

In recent years, China suffered from the deterioration of air quality with the development of rapid urbanization and industrialization [1,2]. Fine particulate matter with an aerodynamic diameter less than 2.5 μm (PM_2.5_) was a major component of the severe air pollution [3,4]. Many epidemiological studies showed that PM_2.5_ is associated with various adverse human health effects, such as respiratory problems and cardiovascular diseases, and can penetrate into human lungs and bronchi [5,6,7,8,9]. Therefore, it is urgent to achieve highly accurate estimates of PM_2.5_ concentrations to effectively assess air quality and conduct health-related studies.

Aerosol optical depth (AOD) can be retrieved from satellite remote sensing, and PM_2.5_ can be estimated based on the correlation between AOD and PM_2.5_. Many satellite instruments, such as the Moderate Resolution Imaging Spectroradiometer (MODIS), the multi-angle imaging spectroradiometer (MISR), the Medium Resolution Spectrum Imager (MERSI), and the Advanced Himawari Imager (AHI), have set bands to retrieve AOD [10,11,12,13,14]. Scholars proposed and improved a variety of methods, such as simple linear regression models, linear mixed effect (LME) models, geographical weight (GWR) models, and machine-learning methods, to establish the relationship between ground-level PM_2.5_ and AOD [15,16,17,18]. Jun Wang et al. used a simple linear model to explore the relationship between AOD and PM_2.5_ in Jefferson County, Alabama, for 2002, and obtained an *R* value of 0.7 [19]. Lee et al. used the LME model to estimate PM_2.5_ in the New England region for 2003 and obtained an *R*^2^ value of 0.92 [20]. Hu el al. developed a GWR model to explore the relationships among PM_2.5_, AOD, meteorological parameters, and land-use information [21]. The results indicated that the GWR model obtained highly accurate estimates of PM_2.5_. Lary et al. combined AOD, meteorological data, and PM_2.5_ to estimate daily PM_2.5_ values from 1997 to 2014 using a machine-learning algorithm [22]. However, it was impossible to retrieve AOD from some regions that were covered by clouds; thus, PM_2.5_ could not be estimated.

Many scholars performed studies to realize ground-level PM_2.5_ concentration estimates with full spatial coverage. Xiao et al. presented a multiple imputation (MI) method that combined the multiangle implementation of atmospheric correction (MAIAC) AOD with community multiscale air quality (CMAQ) simulations to fill in missing AOD values, and they used a two-stage statistical model to estimate the daily ground PM_2.5_ concentrations in the Yangtze River Delta from 2013 to 2014 [23]. Fengchao Liang et al. combined kriging with an external drift (KED) with the Weather Research and Forecasting model with a chemistry module (WRF-Chem) to estimate the daily spatial cover of PM_2.5_ in north China in 2013 [24]. Lv et al. employed a Bayesian-based statistical downscaler to estimate the PM_2.5_ concentrations over the Beijing–Tianjin–Hebei (BTH) region in 2014 and fused the results with the estimated results of atmospheric models [25]. Tao et al. developed a three-stage model by combining ground measurements of PM_2.5_, satellite-derived AOD, and CMAQ data to estimate the daily PM_2.5_ with a spatial resolution of 0.1° over China [26]. However, most studies directly use AOD or only fill missing AOD values before fitting the model to estimate PM_2.5_. Several studies researched fusing different AOD values to improve the utilization of satellite AOD before PM_2.5_ estimation, as well as the use of the nested air-quality prediction modeling system (NAQPMS) for full-coverage PM_2.5_ estimation.

**Figure 1:**
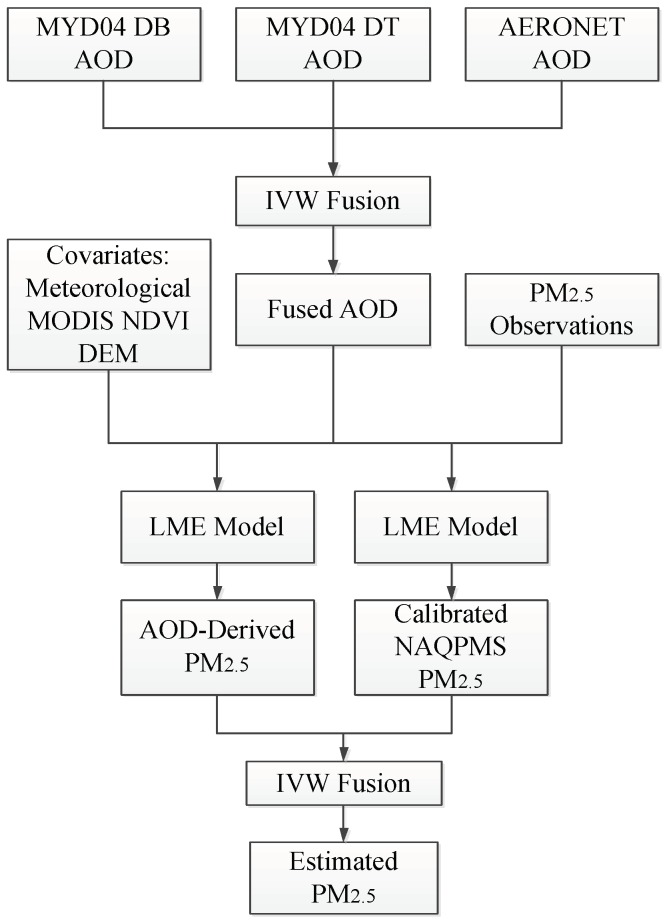
Flowchart of process used to estimate fine ambient particulate matter (PM_2.5_).

The objective of this study was to estimate highly accurate ground-level PM_2.5_ with full spatial coverage in Beijing–Tianjin–Hebei (BTH) using ground monitoring data, MODIS AOD, and atmospheric chemical model data (NAQPMS). Firstly, MODIS dark target (DT) and deep blue (DB) AOD data were fused using the inverse variance weighting (IVW) method to improve the utilization of AOD. Secondly, the LME model was used to estimate PM_2.5_, and the cross-validation (CV) was tested for potential overfitting. Finally, satellite-estimated and calibrated NAQPMS PM_2.5_ data were fused by IVW to obtain the full-coverage spatial distribution of PM_2.5_ in BTH. The flowchart of this process is shown in Figure 1.

## 2. Materials and Methods

### 2.1. Study Area

**Figure 2:**
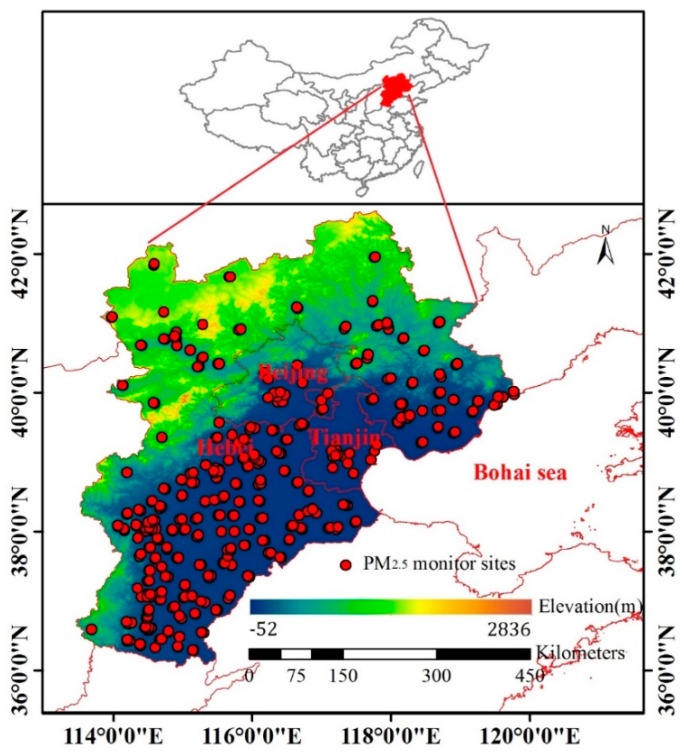
Study area.

The BTH region is located in northern China (Figure 2), which is one of the most economically developed, populated, and polluted regions in the country. Emissions, including coarse and fine particles, mainly come from heavy industries [27,28].

According to the statistical analyses of the China Environmental Bulletin in 2015 and 2016, BTH contained seven and six, respectively, of the top 10 cities with poor air quality among the 74 major cities in China. The annual average concentrations of PM_2.5_ were 77 μg/m^3^ and 71 μg/m^3^ in 2015 and 2016, respectively, and the cities with the worst pollution were Baoding (107 μg/m^3^) and Hengshui (99 μg/m^3^). The air quality in 2017 was better than that in 2016, and the annual average concentration of PM_2.5_ in 2017 was 64 μg/m^3^. The air quality in BTH improved in recent years, but the air pollution problem is still severe. Therefore, it is of great significance to study the concentrations of PM_2.5_, which is the primary pollutant in BTH.

### 2.2. Data Description

#### 2.2.1. PM_2.5_ Data

The hourly PM_2.5_ data used in this study were collected for 2017 from China Environmental Monitoring Center (CNEMC) sites and the Hebei Province Environmental Monitoring Center. PM_2.5_ was mainly monitored using a ThermoFisher TEOM 1405F. The working principle of this instrument is that the air is sampled at a constant flow rate (16.67 L/min), and the mass concentrations of PM_2.5_ are measured by a filter dynamic measurement system and tapered element oscillatory microbalance [29]. A total of 365 sites were investigated in the study, and the locations of all sites are shown in Figure 2. PM_2.5_ values greater than 1000 μg/m^3^ were removed to avoid the effect of outliers, and data with a relative humidity greater than 95% were removed because high relative humidity is produced by rainfall, which can affect the accuracy of pollutant concentration monitoring.

#### 2.2.2. MODIS AOD

The MODIS instrument is carried on both the Terra and Aqua satellites and has a viewing swath width of 2330 km; moreover, this instrument can view the entire surface of the Earth every one to two days [30]. MODIS includes 36 spectral bands (0.405–14.385 μm) with spatial resolutions of 0.25, 0.5 and 1 km [31]. MODIS products are free to download from the official network (http://ladsweb.nascom.nasa.gov/). In this study, MYD04L2 data were downloaded from January to December 2017. The MODIS C6 AOD products mainly included the DT and DB algorithms [32,33]. The basic principle of the DT algorithm is that the land surface reflectances of red (0.66 μm) and blue (0.47 μm) bands are low over dark surfaces (such as dense vegetation), and there is a fixed linear relationship with the 2.1-μm band. However, the DT algorithm fails to retrieve AOD over areas of high reflectivity. The DB algorithm uses a “deep blue” band to distinguish atmospheric and surface contributions and determines the land surface reflectance according to an a priori established surface reflectance database. This approach compensates for the inability of the DT algorithm to retrieve AOD over the bright land surfaces. Because of the characteristics of the two algorithms, the MODIS official website provides the C6 version of the fusion product, which is named “AOD_550_Dark_Target_Deep_Blue_Combined”, to improve the AOD coverage. However, it is yet to be effectively verified, and further improvements are needed in the future [34].

#### 2.2.3. NAQPMS Data

PM_2.5_ simulations from the nested air-quality prediction modeling system (NAQPMS) during 2017 over the BTH region were used in this study. NAQPMS is a multiscale air-quality model developed by the Institute of Atmospheric Physics, Chinese Academy of Sciences. The meteorological conditions inputted to NAQPMS are derived by the WRF model V3.5.1 [35,36]. The WRF model is a mesoscale meteorological model that was jointly developed by the National Centers for Environmental Prediction (NCEP) and the National Center for Atmospheric Research (NCAR). The WRF is widely used to simulate weather phenomena with horizontal resolutions of 1–30 km [37]. The NAQPMS includes real-time emissions, diffusion, aerosol, dry and wet deposition, advection, gaseous phase, aqueous phase, and heterogeneous atmospheric chemical reaction modules [38]. Vertically, the PM_2.5_ simulations include 20 terrain-following layers, and the lowest layer was used in this study. To be consistent with the spatial resolution of the MODIS AOD data, the hourly 9-km PM_2.5_ simulations were resized to 10 km.

#### 2.2.4. Auxiliary Data

Aerosol Robotic Network (AERONET) AOD products (version 3) were collected to fuse DT and DB AOD, and the products were used to validate the fused result. The AERONET AOD includes three levels: Level 1.0 (unscreened), Level 1.5 (cloud-screened and quality controlled), and Level 2.0 (quality-assured) [39]. MODIS AOD data were validated by AERONET level 2.0 observed at three AERONET stations: Beijing (116.38° E, 39.97° N), Beijing_CAMS (116.32° E, 39.93° N), and Xianghe (116.96° E, 39.75° N). To match the AOD at the 0.550-μm band of MODIS, the AODs at 0.440 μm and 0.675 μm were selected to perform an interpolation using the Angstrom exponential [40]. The relative humidity (RH), temperature (TMP), wind speed (WS), and boundary layer height (BLH) were collected from the results of the NAQPMS simulations, with a spatial resolution of 9 km and a temporal resolution of 1 h. The normalized difference vegetation index (NDVI) (MYD13A3), with a spatial resolution of 1 km and a monthly temporal scale, was downloaded from the EOSDIS website (https://search.earthdata.nasa.gov). The ground elevation data came from the geospatial dataset website (http://srtm.csi.cgiar.org/) and had a spatial resolution of 90 m. All of the obtained data were processed at 10 km to be consistent with the spatial resolution of the MODIS AOD data.

### 2.3. Methods Description

#### 2.3.1. Inverse Variance Weighting (IVW) Fusion

MYD04 AOD, including all quality flags (QA = 1, 2, 3), was used to estimate PM_2.5_ to improve the coverage of satellite data. Firstly, a simple linear regression was fitted to define the relationship between DB AOD and DT AOD, which is a method that was used with different satellite instruments and has obtained satisfactory results [1]. The regression equations are shown below.

(1) $$\tau_{\mathrm{DB}}=-0.0065+0.8465\times \tau_{\mathrm{DT}},$$

(2) $$\tau_{\mathrm{DT}}=0.0614+0.9674\times \tau_{\mathrm{DB}},$$

where $\tau_{\mathrm{DB}}$ is DB AOD, $\tau_{\mathrm{DT}}$ is DT AOD, and the *R*^2^ between DB and DT is 0.82. The regression equation was used to fill any grid cell that had only one of the MODIS AODs.

After filling the missing AODs of DT (DB), the AERONET AOD was averaged to within ±30 min of the overpass time of the satellite, and the MODIS AOD (DT and DB) was averaged within a 30-km-radius area at each AERONET station. The difference between the DT (DB) AOD and AERONET AOD was calculated in different seasons. In addition, the variance of the difference was calculated. A large variance indicates that the quality of the satellite AOD data is poor, and the satellite AOD data should be given a lower weight. Conversely, if the variance is small, the satellite AOD data should be given a higher weight [41]. Therefore, the IVW method can be expressed as Equation (3).

(3) $$\tau_{f}=\frac{\frac{\tau_{\mathrm{DB}-f}}{Var_{\mathrm{DB}m}}+\frac{\tau_{\mathrm{DT}-f}}{Var_{\mathrm{DT}m}}}{\frac{1}{Var_{\mathrm{DB}m}}+\frac{1}{Var_{\mathrm{DT}m}}},$$

where $\tau_{\mathrm{DB}-f}(\tau_{\mathrm{DT}-f})$ is the DB (DT) AOD after filling the data, ${Var}_{\mathrm{DB}m}\left({Var}_{\mathrm{DT}m}\right)$ is the variance between DB (DT) AOD and AERONET in season *m*, and $\tau_{f}$ is the IVW-fused AOD. The same procedure was applied to fuse the satellite-estimated PM_2.5_ and the calibrated NAQPMS PM_2.5_.

#### 2.3.2. Model Description

The relationship between ground-level PM_2.5_ and AOD is affected by various factors, such as meteorology and land surface conditions, and varies every day. A simple linear model cannot accurately reflect this relationship. Considering the daily variations in the PM_2.5_–AOD relationship, Lee et al. (2011) proposed the LME model to estimate PM_2.5_ [20]. Many scholars constructed the model by adding various meteorological parameters, land-use data, population densities, and other parameters for different regions and different times. The results of these studies suggest that added parameters improve the accuracy of PM_2.5_ estimates [42].

In this study, the LME model was used to estimate PM_2.5_ with MODIS AOD, meteorological factors, NDVI, and elevation, which can be expressed as Equation (4). In addition, the NAQPMS PM_2.5_ values were calibrated by the LME model, which is expressed as Equation (5).

(4) $$\begin{array}{l} {\mathrm{PM}}_{2.5,st}=\left(\alpha +\omega_{t}\right)+\left(\beta_{1}+\mu_{1,t}\right)\times {\mathrm{AOD}}_{st}+\left(\beta_{2}+\mu_{2,t}\right)\times {\mathrm{TMP}}_{st}+\left(\beta_{3}+\mu_{3,t}\right)\times {\mathrm{RH}}_{st}\\ \;+\left(\beta_{4}+\mu_{4,t}\right)\times {\mathrm{WS}}_{st}+\left(\beta_{5}+\mu_{5,t}\right)\times {\mathrm{BLH}}_{st}+\left(\beta_{6}+\mu_{6,j}\right)\times {\mathrm{NDVI}}_{sj}\\ \;+\beta_{7}\times {\mathrm{ELEV}}_{s}+\varepsilon , \end{array}$$

(5) $${\mathrm{PM}}_{2.5,st}=(\alpha^{\mathrm{NAQPMS}}+\omega_{t}^{\mathrm{NAQPMS}})+\left(\beta_{1}^{\mathrm{NAQPMS}}+\mu_{1,t}^{\mathrm{NAQPMS}}\right)\times {\mathrm{NAQPMS}}_{st}+\varepsilon ,$$

where ${\mathrm{PM}}_{2.5,st}$ is the ground-level PM_2.5_ measurement (μg/m^3^) at site *s* on day *t*; α and $\omega_{t}$ (day-specific) are the fixed and random intercepts, respectively; ${\mathrm{AOD}}_{st}$ represents the MODIS-fused AOD (unitless) at site *s* on day *t*; and $\beta_{1}$ and $\mu_{1,t}$ (day-specific) are the fixed and random slopes for the AOD predictor, respectively. ${\mathrm{TMP}}_{st},\;{\mathrm{RH}}_{st},\;{\mathrm{WS}}_{st},\;\mathrm{and}\;{\mathrm{BLH}}_{st}$ are the meteorological factors that represent TMP (°C), RH (%), WS (m/s), and BLH (m), respectively, at site *s* on day *t*; $\beta_{2}\sim \beta_{5}$ and $\mu_{2,t}\sim \mu_{5,t}$ are the fixed slopes and random day-specific slopes, respectively, for the meteorological predictors; ${\mathrm{NDVI}}_{\mathrm{sj}}$ and ${\mathrm{ELEV}}_{s}$ represent the NDVI (unitless) at site s in month j and the ground elevation (m) at site s, respectively; $\beta_{6}\sim \beta_{7}$ and $\mu_{6,j}$ are the fixed slopes and random month-specific slope, respectively; and ε represents the residual error. In Equation (5), ${\mathrm{PM}}_{2.5,\mathrm{st}}$ is the same as that in Equation (4), and ε represents the residual error; $\alpha^{\mathrm{NAQPMS}}$ and $\omega_{t}^{\mathrm{NAQPMS}}$ are the fixed intercept and random day-specific intercept, respectively; ${\mathrm{NAQPMS}}_{st}$ is the PM_2.5_ concentration (μg/m^3^) simulated by NAQPMS; and $\beta_{1}^{\mathrm{NAQPMS}}$ and $\mu_{1,t}^{\mathrm{NAQPMS}}$ are the fixed slope and random day-specific slope, respectively, for NAQPMS PM_2.5_.

#### 2.3.3. Model Validation

To test for potential overfitting of the model, a 10-fold cross-validation (CV) method was selected in this study. All of the samples were first split into ten folds, with approximately 10% of the total data in each fold. In each instance of CV, one of the folds was used for testing samples, and the remaining nine folds were used to fit the model [17]. The predicted values of the testing samples made from the fitted model were recorded. This process was repeated ten times until all of the folds were used. The agreements between the predicted PM_2.5_ concentrations from the 10-fold CV and the measured PM_2.5_ concentrations were evaluated using the coefficient (*R*^2^), mean prediction error (MPE), and root-mean-square error (RMSE).

## 3. Results and Discussion

### 3.1. Descriptive Statistics

**Figure 3:**
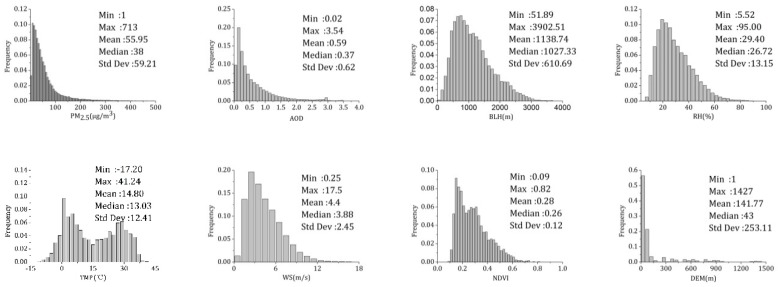
Histograms and descriptive statistics (minimum, maximum, mean, median, and standard deviation values) of dependent and independent variables for the Moderate Resolution Imaging Spectroradiometer (MODIS).

The histograms and statistics of the variables are illustrated in Figure 3. All variables are approximately unimodal and log-normally distributed, except for temperature, and the frequency distribution of PM_2.5_ and AOD is basically consistent. The mean, minimum, and maximum PM_2.5_ concentrations were 55.95 μg/m^3^, 1 μg/m^3^, and 713 μg/m^3^, respectively. The mean, minimum, and maximum AOD values were 0.59, 0.02, and 3.54, respectively. Both the maximum values of PM_2.5_ and the maximum values of AOD were high, indicating that there was severe pollution during the study period. The average BLH was 1138.74 m. The lower average RH (29.40%) indicated that the atmospheric environment was relatively dry in BTH, which was related to the geographical location of the study area. The fluctuation of WS was large, with a range of 0.25–17.5 m/s and an average WS of 4.4 m/s. The TMP had a typical seasonal variation and showed a bimodal distribution, with a range of −17.20–41.24 °C. The average elevation of the monitoring sites was 141.77 m, and the standard deviation of the elevation was 253.11 m. The variation range of the NDVI was 0.09–0.82, with an average value of 0.28, and the NDVI was concentrated between 0.1 and 0.4.

### 3.2. Validation of Fused MODIS AOD

**Figure 4:**
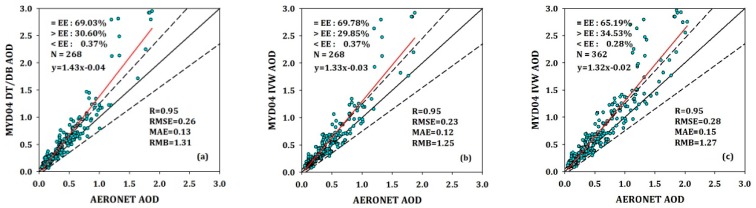
Scatterplots and statistical parameters of dark target (DT) and deep blue (DB) aerosol optical depth (AOD) (**a**), Method I (**b**), and Method II (**c**). The dashed lines represent the expected error (EE), the black solid line represents the 1:1 line, and the red line represents the fitted linear regression line.

The fused MODIS AOD produced using the IVW method was validated against the AERONET AOD. The Spearman correlation coefficient (*R*), mean absolute error (MAE), RMSE, relative mean bias (RMB), and expected error (EE) were calculated to evaluate the accuracy of the AOD fusion [39]. The EE used in this study was ±(0.03 + 0.2AOD_AERONET_), which was the same as the EE of the DB algorithm over land. Figure 4 shows the scatterplots and statistical parameters between the MODIS AOD and AERONET AOD. Figure 4a shows the MYD04 AOD extracted from the MODIS office products (“AOD_550_Dark_Target_Deep_Blue_Combined” as DT and DB), Figure 4b shows the fused AOD produced using the IVW method at the same matched samples as DT and DB (i.e., the same as Method I), and Figure 4c shows the fusion AOD produced using the IVW method (i.e., the same as Method II). The fusion result of Method I outperformed that of DT and DB, with a lower RMSE (~0.23) and MAE (~0.12), and ~69.78% of the samples fell within the EE, with 29.85% and 0.37% of the collections being overestimated and underestimated, respectively, indicating little aerosol estimation uncertainty (RMB = 1.25). The fusion result of Method II added 94 AOD samples, and the number of samples with AOD > 1.0 increased significantly. Furthermore, the samples falling within the EE decreased by 3.84%, RMSE increased by 7.7%, MAE increased by 0.02, and RMB was closer to 1 (decreased by 0.04), indicating that the fusion results were viable.

**Figure 5:**
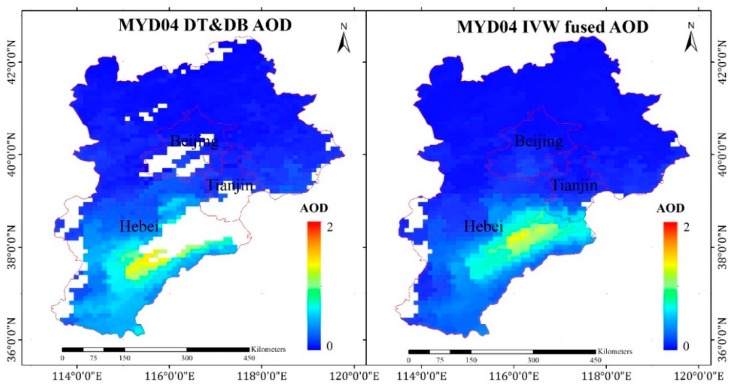
AOD distribution in Beijing–Tianjin–Hebei (BTH) (6 September 2017).

In terms of the spatial distribution, the fusion result of Method II had higher AOD coverage than that of DT and DB AOD on 6 September 2017, as shown in Figure 5. The DT and DB AOD had many missing data, especially for high-value AODs, which was caused by the fusion method. Therefore, according to the validation and spatial distribution of AOD, Method II had a high accuracy and greater coverage, which was conducive to estimating the PM_2.5_ concentrations in this study.

### 3.3. Model Validation

#### 3.3.1. MODIS-AOD-Estimated PM_2.5_

**Figure 6:**
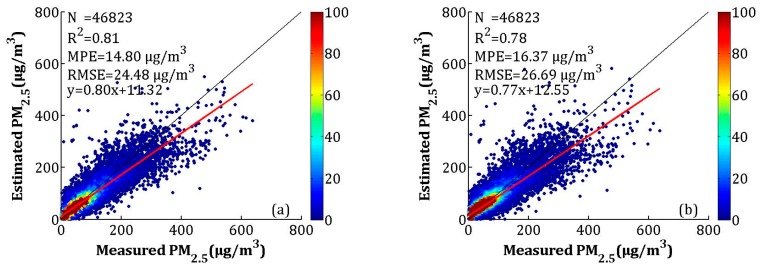
Scatterplots between the linear mixed effect (LME) model fitting (cross-validation) and ground-measured PM_2.5_: (**a**) the model fitting result, and (**b**) the cross-validation results. The dark solid line represents the 1:1 line, and the red solid line represents the regression line.

The LME model fitting result and the CV are shown in Figure 6a,b, respectively. According to the scatterplots of the model fitting results (Figure 6a), the LME model explained 81% of the variability in the ground-level PM_2.5_ concentrations, with an *R*^2^ of 0.81. The MPE, RMSE, and slope were 14.80 μg/m^3^, 24.48 μg/m^3^, and 0.80, respectively. Compared to the model fitting results, the *R*^2^ and slope of CV decreased by 0.03, and the MPE and RMSE increased by 1.57 and 2.21 μg/m^3^, respectively, which showed that the LME model had overfitting. However, both the model fitting and CV had high *R*^2^ values, which can indicate the relationship between AOD and PM_2.5_.

**Figure 7:**
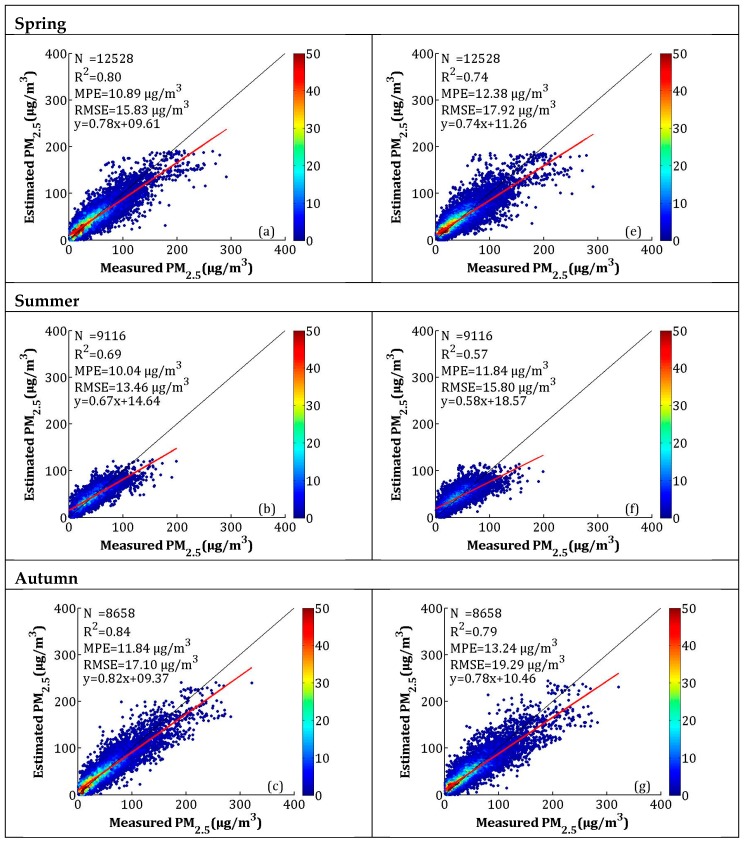

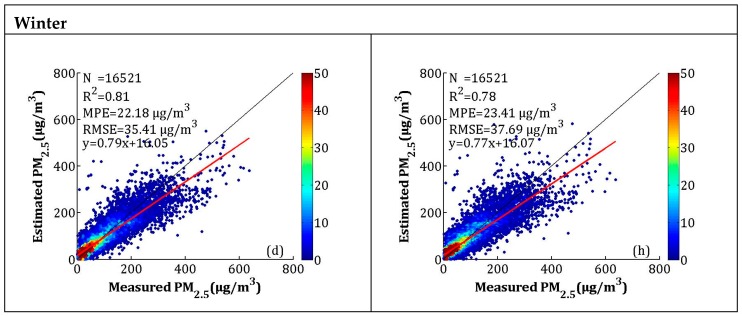
Scatterplots between the LME model fitting (cross-validation) and ground-measured PM_2.5_ in each season: (**a**–**d**) the LME model fitting results, and (**e**–**h**) the cross-validation results. The dark solid line represents the 1:1 line, and the red solid line represents the regression line.

The seasonal model fitting and CV results are shown in Figure 7a–h. According to Figure 7a–d, the seasonal model fitting *R*^2^ values for spring (i.e., March, April, and May), summer (i.e., June, July, and August), autumn (i.e., September, October, and November), and winter (i.e., December, January, and February) were 0.80, 0.69, 0.84, and 0.81, respectively. The MPEs were 10.89, 10.04, 11.84, and 22.18 μg/m^3^, respectively. The RMSEs were between 13.46 and 35.41 μg/m^3^, and the slopes were between 0.67 and 0.82. The *R*^2^ of the CV in spring, summer, autumn, and winter decreased by 0.06, 0.12, 0.05, and 0.03, respectively, while the MPEs respectively increased by 1.49, 1.80, 1.40, and 1.23 μg/m^3^, and the RMSEs respectively increased by 2.09, 2.34, 2.19, and 2.28 μg/m^3^. The model fitting and CV results showed that the LME performed best in autumn and worst in summer. According to Figure 6 and Figure 7, the high values (>300 μg/m^3^) of the PM_2.5_ concentrations were obviously underestimated, which may be related to the larger uncertainty in the estimated AOD during hazy weather and dust storms [43]. A previous study showed that large errors in AOD retrieval occur when AOD levels are extremely high [30].

#### 3.3.2. Calibrated NAQPMS PM_2.5_

**Figure 8:**
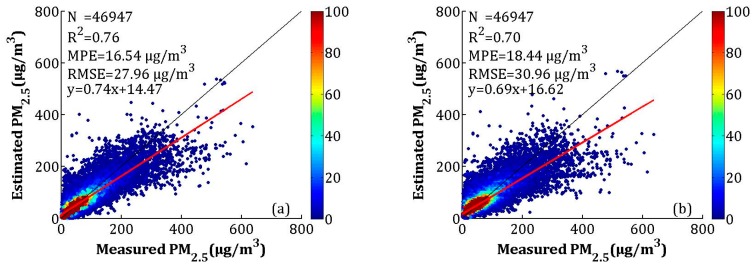
Scatterplots between the LME model fitting (cross-validation) by the nested air-quality prediction modeling system (NAQPMS) data and ground-measured PM_2.5_ in each season: (**a**) the LME model fitting results, and (**b**) the cross-validation results. The dark solid line represents the 1:1 line, and the red solid line represents the regression line.

To improve the accuracy of the estimated PM_2.5_, the NAQPMS data were calibrated using the LME model, which is described in Section 2.3.2. The model fitting and CV of the overall data are shown in Figure 8. The *R*^2^, MPE, and RMSE of the model fitting were 0.76, 16.54, and 27.96 μg/m^3^, respectively. The CV result suggested that the model had overfitting, which was similar to the AOD-PM_2.5_ model performance. The *R*^2^ decreased by 0.06, and the MPE and RMSE increased by 1.90 and 3.00 μg/m^3^, respectively.

**Figure 9:**
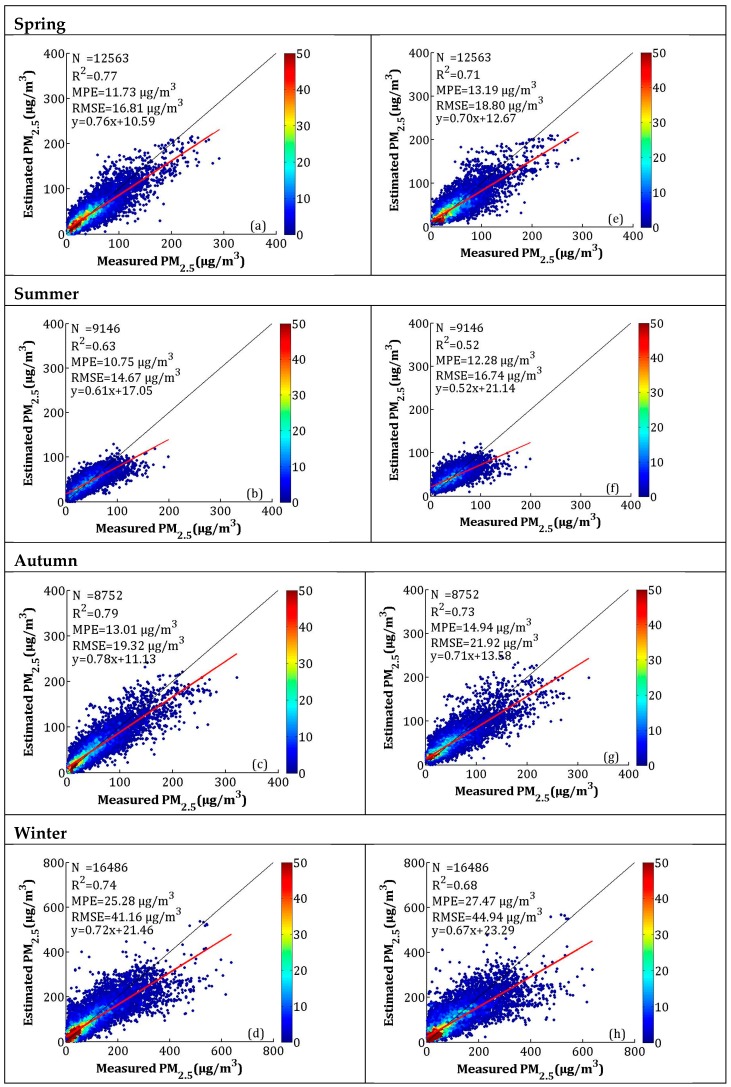
Scatterplots between the LME model fitting (cross-validation) by NAQPMS data and ground-measured PM_2.5_ in each season: (**a**–**d**) the LME model fitting results, and (**e**–**h**) the cross-validation results. The dark solid line represents the 1:1 line, and the red solid line represents the regression line.

The scatterplots and CV for the seasonal model fitting are presented in Figure 9. The *R*^2^ values of model fitting in spring (MAM), summer (JJA), autumn (SON), and winter (DJF) were 0.77, 0.63, 0.79, and 0.74, respectively. The MPEs for spring, summer, autumn, and winter were 11.73, 10.75, 13.01, and 25.28 μg/m^3^, respectively, and the RMSEs were 16.81, 14.67, 19.32, and 41.16 μg/m^3^, respectively. The *R*^2^ of CV decreased by 0.06, 0.11, 0.06, and 0.06, the MPEs increased by 1.46, 1.53, 1.93, and 2.19 μg/m^3^, and the RMSEs increased by 1.99, 2.07, 2.60, and 3.78 μg/m^3^ in spring, summer, autumn, and winter, respectively. Similar to the AOD-PM_2.5_ model fitting and CV results, the NAQPMS PM_2.5_ calibration results also showed underestimation when PM_2.5_ concentrations were high. This phenomenon can be explained as follows: during heavy pollution, haze affects the BLH, lowering the BLH and slowing the WS near the ground, which results in the accumulation of pollution. However, the air-quality model cannot adequately capture this process and, thus, causes underestimations [37].

#### 3.3.3. Fused PM_2.5_ Validation

**Figure 10:**
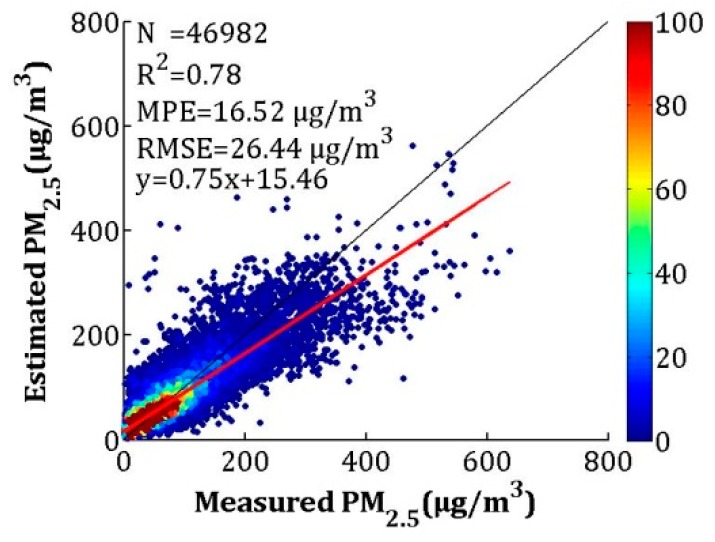
Assessment of overall LME model performance based on measured and estimated PM_2.5_ concentrations for cross-validation.

**Figure 11:**
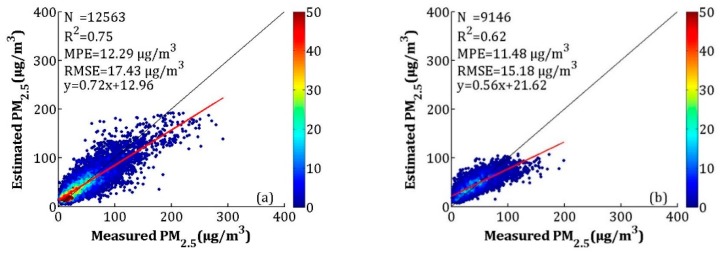

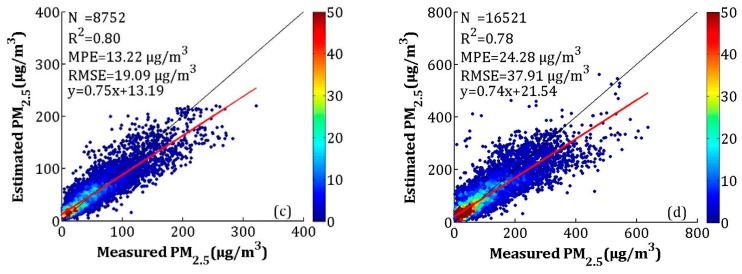
Assessment of seasonal LME model performance based on measured and estimated PM_2.5_ concentrations for cross-validation (**a**) spring, (**b**) summer, (**c**) autumn, (**d**) winter.

To make full use of the high accuracy of the satellite-estimated PM_2.5_ and the full coverage of NAQPMS-calibrated PM_2.5_, the IVW method was used in this study. The overall and seasonal CV results are shown in Figure 10 and Figure 11, respectively. For the overall validation, the *R*^2^, MPE, and RMSE values were 0.78, 16.52 and 26.44 μg/m^3^, which were close to the CV results of satellite-estimated PM_2.5_ (*R*^2^ = 0.78, MPE = 16.37 μg/m^3^, and RMSE = 26.69 μg/m^3^) and better than the calibrated NAQPMS PM_2.5_ results (*R*^2^ = 0.70, MPE = 18.44 μg/m^3^, and RMSE = 30.96 μg/m^3^). For the seasonal validation results, the *R*^2^ values for spring, summer, autumn, and winter were 0.75, 0.62, 0.80, and 0.78, respectively. The MPEs were 12.29, 11.48, 13.22 and 24.28 μg/m^3^, respectively, and the RMSEs were 17.43, 15.18, 19.09 and 37.91 μg/m^3^ in the spring, summer, autumn, and winter, respectively. The PM_2.5_ fusion results were more realistic than the satellite-estimated PM_2.5_ and the calibrated NAQPMS PM_2.5_, except for winter. For the fusion result, the *R*^2^ values increased between 0.01 and 0.10, the MPEs decreased between 0.02 and 1.72 μg/m^3^, and the RMSEs decreased between 0.20 and 2.83 μg/m^3^ in spring, summer, and autumn.

**Figure 12:**
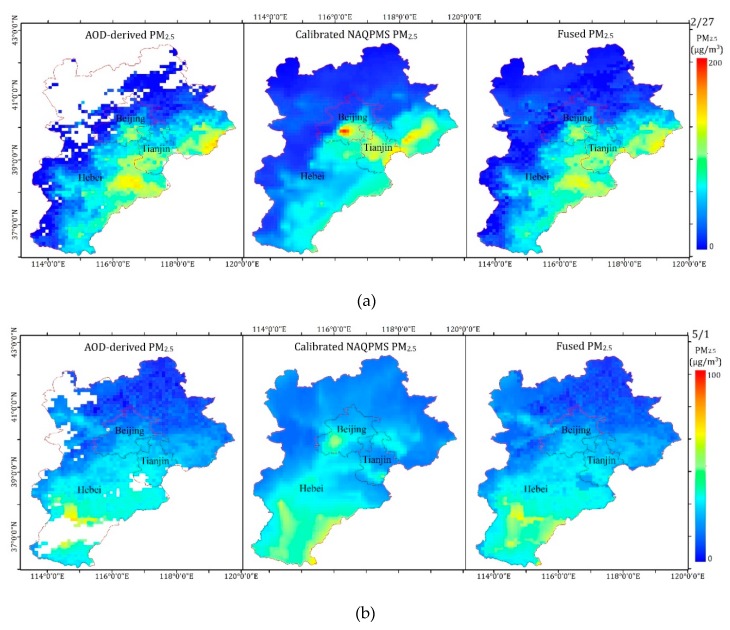

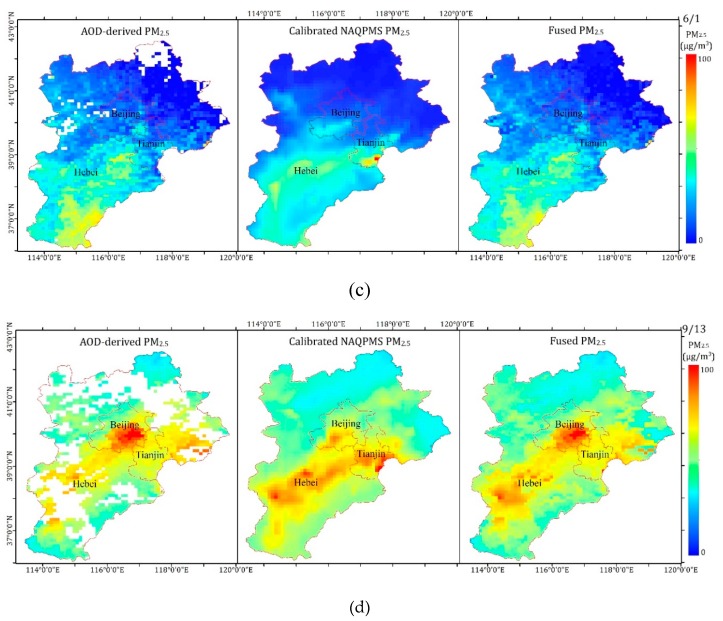
Spatiotemporal distributions of AOD-derived PM_2.5_, calibrated NAQPMS PM_2.5_, and fused PM_2.5_. (**a**) 27 February 2017, (**b**) 1 May 2017, (**c**) 1 June 2017, and (**d**) 13 September 2017.

The spatiotemporal distributions of AOD-derived PM_2.5_ (left), calibrated PM_2.5_ (center), and fused PM_2.5_ (right) in the BTH region are shown in Figure 12. As shown in the figure, the results obtained by the three methods showed the same spatial distribution for concentrations of PM_2.5_. The concentrations of PM_2.5_ were higher in the southern BTH region and lower in the northern BTH region. Heavy pollution was concentrated in densely populated urban areas, and mountainous areas were less polluted. According to Figure 12, the satellite-estimated PM_2.5_ missed some pollution information due to incomplete coverage. For instance, a large number of pixels were missing in the northwestern BTH region due to the influence of clouds, ice, and snow on 27 February 2017. The losses of pixels on 1 May, 1 June, and 13 September 2017 were mainly due to the influence of clouds. Therefore, it was impossible for the data to reflect the complete pollution process. The calibrated NAQPMS PM_2.5_ achieved full spatial coverage, but the results were overly smoothed, which was affected by emission inventory and system errors. In addition, overestimation or underestimation in some regions occurred in this study. For example, the average ground-measured PM_2.5_ was 56.76 μg/m^3^ on 27 February 2017, in Beijing, but the calibrated NAQPMS PM_2.5_ value in Beijing was 76.88 μg/m^3^. Unlike them, the fused PM_2.5_ obtained a better result by considering the advantages of high accuracy, detailed information, and full coverage. The annual mean ground-level PM_2.5_, fused PM_2.5_, satellite-estimated PM_2.5_, and calibrated NAQPMS PM_2.5_ values at the same sites were 55.95, 55.42, 55.04 and 54.42 μg/m^3^, respectively, indicating that the fused PM_2.5_ was best.

## 4. Conclusions

MODIS AOD, ground-measured PM_2.5_, and NAQPMS PM_2.5_ were used to perform the LME model and IVW fusion in this study; this method overcame the influences of cloud, ice, and snow and obtained highly accurate PM_2.5_ estimates with full spatial coverage. Before PM_2.5_ estimation, the IVW method was used to increase the coverage of AOD. According to the verification and analysis results, the AOD fused using the IVW method had a high accuracy with a high correlation (*R* = 0.95) and a low RMSE (0.28), and the coverage of AOD increased significantly. The results indicated that the AOD fused using the IVW method was more suitable than the non-fused AOD for estimating PM_2.5_ concentrations. The fused PM_2.5_ in this study was highly correlated with the ground-level measurements and yielded a CV *R*^2^ of 0.78; this value was the same as the CV *R*^2^ of the satellite-estimated PM_2.5_ and outperformed the CV *R*^2^ of the calibrated NAQPMS PM_2.5_ (CV *R*^2^ = 0.70). In addition, the PM_2.5_ estimation results showed obvious seasonal differences, and the CV *R*^2^ values of each season were 0.75, 0.62, 0.80, and 0.78. Therefore, the fusion results can supplement the lack of information on satellite-estimated PM_2.5_ and capture more detailed information than the calibrated NAQPMS PM_2.5_. The results can be used for analyzing pollution processes, as well as for health-related studies.
